# Laboratory study of stationary accretion shock relevant to astrophysical systems

**DOI:** 10.1038/s41598-019-44596-3

**Published:** 2019-05-31

**Authors:** P. Mabey, B. Albertazzi, E. Falize, Th. Michel, G. Rigon, L. Van Box Som, A. Pelka, F.-E. Brack, F. Kroll, E. Filippov, G. Gregori, Y. Kuramitsu, D. Q. Lamb, C. Li, N. Ozaki, S. Pikuz, Y. Sakawa, P. Tzeferacos, M. Koenig

**Affiliations:** 1 LULI - CNRS, Ecole Polytechnique, CEA, Université Paris-Saclay, F-91128 Palaiseau Cedex, France; 2 CEA-DAM-DIF, F-91297 Arpajon, France; 3 CEA Saclay, DSM/Irfu/Service d’Astrophysique, F-91191 Gif-sur-Yvette, France; 4 Helmholtz-Zentrum Dresden-Rossendorf (HZDR), Bautzner Landstr. 400, D-01328 Dresden, Germany; 5 Technische Universität Dresden, D-01062 Dresden, Germany; 6 JIHT-RAS, 13-2 Izhorskaya st., Moscow, 125412 Russia; 7 National Research Nuclear University MEPhI, Moscow, 115409 Russia; 8 Department of Physics, University of Oxford, Parks Road, Oxford, OX1 3PU UK; 9 Graduate School of Engineering, Osaka University, Suita, Osaka, 565-0871 Japan; 10 Department of Physics, National Central University, Taoyuan City, Taiwan; 11 Department of Astronomy and Astrophysics, University of Chicago, Chicago, IL USA; 12 Plasma Science and Fusion Center, Massachusetts Institute of Technology, 77 Massachusetts Avenue, Cambridge, MA 02139 USA; 13 Institute of Laser Engineering, Osaka University, Suita, Osaka, 565-0871 Japan

**Keywords:** Laboratory astrophysics, Laser-produced plasmas, Astrophysical plasmas

## Abstract

Accretion processes play a crucial role in a wide variety of astrophysical systems. Of particular interest are magnetic cataclysmic variables, where, plasma flow is directed along the star’s magnetic field lines onto its poles. A stationary shock is formed, several hundred kilometres above the stellar surface; a distance far too small to be resolved with today’s telescopes. Here, we report the results of an analogous laboratory experiment which recreates this astrophysical system. The dynamics of the laboratory system are strongly influenced by the interplay of material, thermal, magnetic and radiative effects, allowing a steady shock to form at a constant distance from a stationary obstacle. Our results demonstrate that a significant amount of plasma is ejected in the lateral direction; a phenomenon that is under-estimated in typical magnetohydrodynamic simulations and often neglected in astrophysical models. This changes the properties of the post-shock region considerably and has important implications for many astrophysical studies.

## Introduction

Accretion processes are of much interest to the astrophysics community, as they are thought to supply power in various astrophysical objects, as well as being the dominant radiation source in many binary systems^1^. Understanding the complex physical processes that allow the release of gravitational energy in the form of radiation, is fundamental to interpreting high-energy astronomical observations. According to the magnetospheric accretion model, a shock is formed when material from a stellar accretion disk falls down and impacts upon the surface of the star^2–4^. This material is taken away from the plane of the disk and directed along the star’s magnetic field lines and onto its surface. The process is therefore highly dependent on the nature and strength of the star’s magnetic field. There is now a wealth of evidence to support the existence of these accretion shocks across a range of different systems^5,6^.

In the context of young stars, the final mass is ultimately determined by the accretion mechanism during the early stages of its formation and evolution towards main sequence. Therefore the study of accreting young stars and, in particular, the way in which matter settles on the their surface is wide interest in the context of star formation^7^. As for the different binary star systems, magnetic cataclysmic variable stars (MCVs) represent a unique opportunity to perform a focused study on accretion dynamics, since the accretion region is responsible for the majority of the luminosity of these systems. Moreover, MCVs have long been discussed as potential progenitors of type Ia supernovae^8^, and so understanding their dynamics is crucial to explain the initial conditions of these explosions, which themselves are used to study the acceleration of the Universe^9^.

MCVs consist of a strongly magnetised white dwarf (WD) accreting matter from a low-mass companion star^10^. Of particular interest are the subclasses of systems known as polars or intermediate polars, the former being characterised by a single optical and X-ray photometric period and strong optical linear (5–10%) and circular (10–80%) polarisation. Due to the very strong magnetic field of the white dwarf (*B* ~ 0.01–1 MG for intermediate polars and up to *B* > 10 MG for polars), the accreting plasma flow (*υ* ~ 1000 km/s) is guided along the field lines onto the WD photosphere, forming a column rather than an accretion disk^11–13^. After impact on the WD photosphere, a radiative reverse shock is formed, which propagates counter to the incoming flow, thus heating the accretion column to temperatures up to 10 keV. Consequently, an intense spectrum from soft to hard X-rays, predominantly due to bremsstrahlung cooling, is observed. However, the observed ratio of hard to soft X-ray emission currently disagrees with the standard model of accretion columns^14^. Additionally, unexplained luminosity oscillations, possibly related to unstable thermal oscillations in the shock front, or magnetohydrodynamic instabilities in the accretion column, have also been reported^15,16^. The intense radiation emitted in the post-shock region acts to slow down the accretion shock, which reaches a steady height of *h*_*s*_ ~ 100–1000 km above the WD photosphere. However, a complete stoppage of the infalling plasma requires an infinitely large radiative energy density in the lower part of the accretion column^17,18^. The first models of accretion systems therefore invoked singularities in order to ensure the stationarity of the shock^11^. Modern studies, however, suggest the possibility of mass being ejected laterally upon collision with the star’s surface^19–21^, thus maintaining a constant shock height. Many theories relating the properties of the WD (e.g. its mass) to this height^22,23^ have been proposed, but the distance is far too small to be resolved with observations. Therefore, obtaining spatial profiles of the radiative zone, reconciling observations with theory, and ultimately confirming the properties of WDs all remain out of reach.

Small-scale models of accretion columns, based on similarity relations to their astrophysical counterparts, can now be created using high-power lasers^21^. Radiation hydrodynamics permits exact^24,25^ or parametric^26^ scaling laws that permits the comparison of scales in the laboratory to those in astrophysics, with a high degree of fidelity. In this paper, we present a scaled study, built on well established experimental platforms^27–31^ to investigate accretion shocks in binary systems. In this experiment, a strong magnetic field is imposed on the experimental system, capable of collimating the plasma flow as in the astrophysical case. Improvements are thus made on previous work where either the plasma flow was left to expand freely with no collimation mechanism or a tube was employed to artificially collimate the flow, strongly influencing the observations. Various diagnostics are employed, allowing a full understanding of the dynamics of the system, over the full timescale of the experiment. Evidence of a new shock structure is seen, whereby the return shock is initially slowed down by a strongly radiative region upstream and remains stationary for a period in excess of 60 ns, caused by lateral mass ejection in the collision region.

## Results

**Figure 1:**
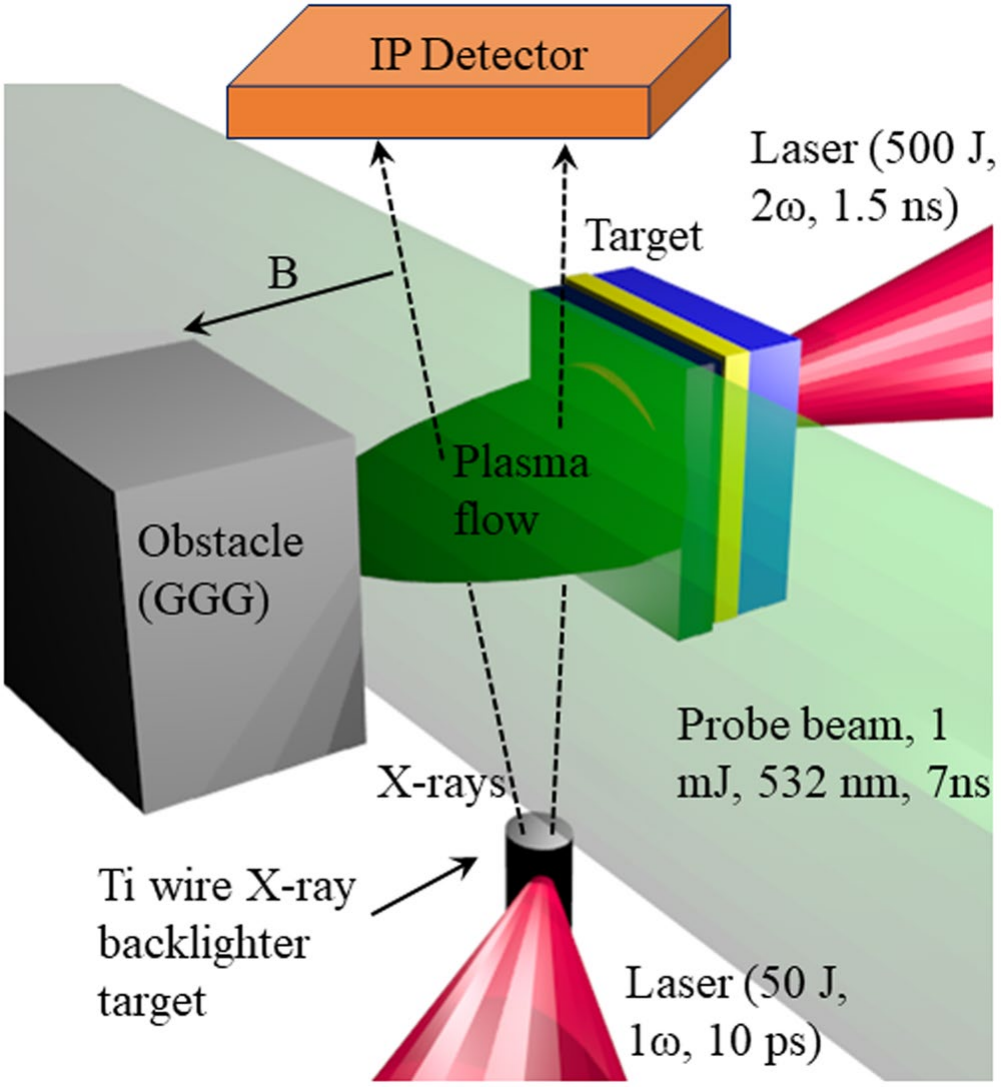
Experimental setup at the LULI2000 laser facility. The drive laser, interacts with the multi-layer target, creating a plasma flow travelling towards the obstacle. The interaction of a short-pulse laser with a wire target generates X-rays which are used to radiograph the plasma flow, with an image plate detector. An optical probe beam, orthogonal to the plasma flow is used to perform Schlieren imaging, and self-emission of the experiment is recorded on the same axis. A constant, homogeneous magnetic field of 15 T in the direction of the plasma flow is applied across the whole system.

Figure 1 displays a schematic of the experiment, which was carried out at the LULI2000 laser facility. A full description is available in the Methods section.

### Initial plasma flow

**Figure 2:**
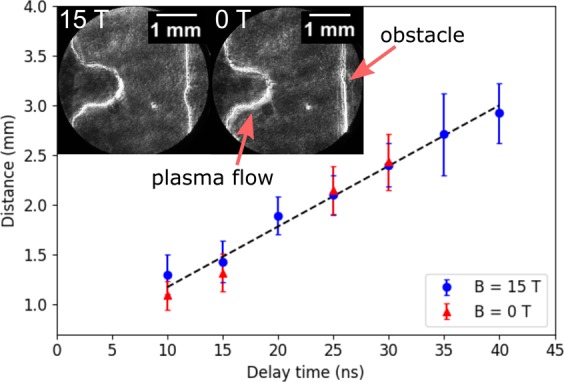
The distance travelled from the target by the plasma flow as measured by the Schlieren imaging system. Two example images are shown with and without an imposed magnetic field respectively, both with a delay time of 10 ns. No differences in speed or plasma structure are observed between the two cases. The estimated plasma flow speed is shown by the straight line on the graph and is measured to be 78 ± 5 km/s.

The evolution of the plasma flow, resulting from the initial laser-target interaction, is measured by means of two gated Schlieren imaging systems. These are sensitive to the electronic density gradients associated with the front of the plasma flow. Their delays are varied independently, with respect to the drive laser, in order to determine the speed of the front of the plasma flow towards the obstacle. The progression of the flow with time is shown in Fig. 2, together with two sample Schlieren images with and without an imposed magnetic field of 15 T, respectively. The speed is thus determined to be 78 ± 5 km/s. This is in agreement with previous results using similar targets and laser conditions^30,32^. The magnetic field does not affect the speed of the plasma flow as expected. The increase in density in the post-shock region renders this diagnostic unsuitable for studying the reverse shock and so delays are limited to the phase of the experiment where the plasma flows towards the obstacle.

**Figure 3:**
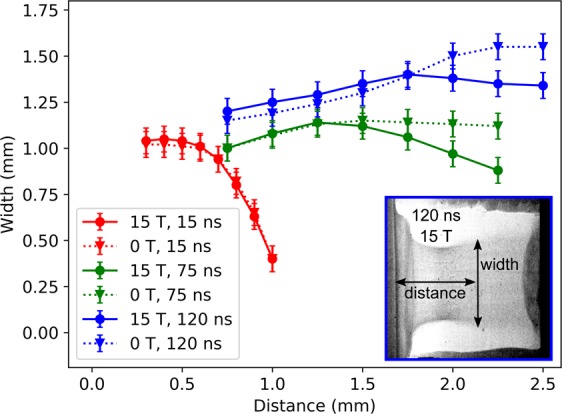
The width of the plasma flow as a function of the distance away from the target, s measured at various delay times with and without an imposed magnetic field. The widths at 15 ns are measured using the Schlieren diagnostic whereas those at 75 ns and 120 ns are measured using the radiography images. At early times, in the flow-launching region, the collimating role of the magnetic is not yet noticeable, while at later times a clear decrease in flow width is observed when the field of 15 T is applied. The inset shows, as an example, the raw data for the X-ray radiography image at 120 ns with an imposed field of 15 T, showing how the measurements are taken.

At higher densities, X-ray radiography is employed to probe the physical structure of the plasma flow as well as that of the reverse shock^33^. Images are taken along the axis orthogonal to the optical diagnostics and the plasma flow. The probe laser, used to generate the short X-ray source, is delayed with respect to the drive laser, to track the progress of the system with time. The collimating effect of the magnetic field on the plasma flow is investigated at early times by the Schlieren images and at late times by the radiography. Figure 3 illustrates that at early times (*t* ~ 15 ns) the effect of the magnetic field is not yet discernible, as the magnetic pressure is small compared with the ram pressure of the plasma. However, at later times, from around *t* ~ 75 ns to *t* ~ 180 ns, the width of the flow is noticeably smaller (~20%) when the magnetic field of 15 T is imposed. This follows the dynamics described by^30^, where, after an initial flow launching region, the magnetic pressure is expected to be sufficient to manipulate the large-scale structure of the flow. One should note also that there is a non-negligible hydrodynamic collimation of the flow, via a nozzle mechanism, even without the imposed magnetic field, as observed in previous work^31^.

**Figure 4:**
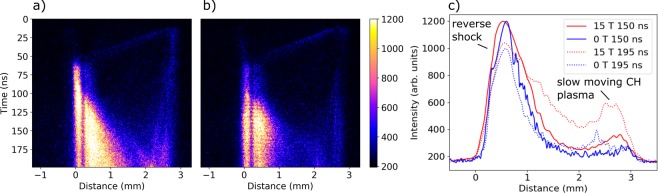
1-D Streaked self-emission of the experimental system. Images show raw data from a single shot with (**a**) an imposed magnetic field of 15 T and (**b**) no imposed magnetic field. In both cases, the laser arrives from the top right-hand side of the image and the plasma initially flows from right to left. A fault in the streak camera used to obtain the data gives rise to a vertical band of insensitive pixels between 0.175 and 0.279 mm from the obstacle. An interpolation algorithm is used in the analysis of this region. (**c**) Horizontal lineouts at 150 and 195 ns averaged averaged over 3 shots for the 0 T case and 6 shots for the 15 T case, showing an increase in emission at late times with the imposed magnetic field.

One-dimensional, streaked, self-emission of the whole experimental system is also obtained and displayed in Fig. 4 for shots with and without the imposed magnetic field. The plasma is created at the upper right hand corner of the panel, at *t* = 0, *x* = 3 mm. It then moves from right to left at a speed of 75 ± 10 km/s towards the obstacle, which is at a distance of 3 mm away from the initial laser target interaction. When the flow impacts upon the obstacle (~60 ns), it heats up and begins to emit. For a short period of time, material builds up on the surface of the obstacle, before eventually a strongly emitting reverse shock is formed, moving counter to the incoming plasma flow. Also seen in both the 2D images and the lineouts is a flow of weakly emitting plasma, moving slowly from the target towards the obstacle. This flow is predominantly made up of the CH ablator layer in the target and does not play a role in the interaction with the obstacle. We note that the speed of the incoming flow is in agreement with that measured by the Schlieren imaging diagnostic.

### Reverse shock

In examining the behaviour of the reverse shock, we first note that the emission is stronger at later times in the case of the imposed magnetic field. This can be explained by recognizing the fact that the density of the plasma flow is higher in the presence of the magnetic field, due to the collimating effect seen in Fig. 3 and described in the previous section. This in turn leads to higher temperatures in the post-shock region also. The interplay of the two effects combined therefore leads to an increase in the emission seen by the detector. To illustrate this point further, Fig. 4c shows horizontal lineouts from the streaked images at 150 and 195 ns, averaged over multiple shots. The difference between the two cases is initially small, whereas at later times, the maximum emission increases by up to a factor of 50%. The small peaks on the right hand side of the graph correspond to the slow moving CH plasma emanating from the multi-layer target.

**Figure 5:**
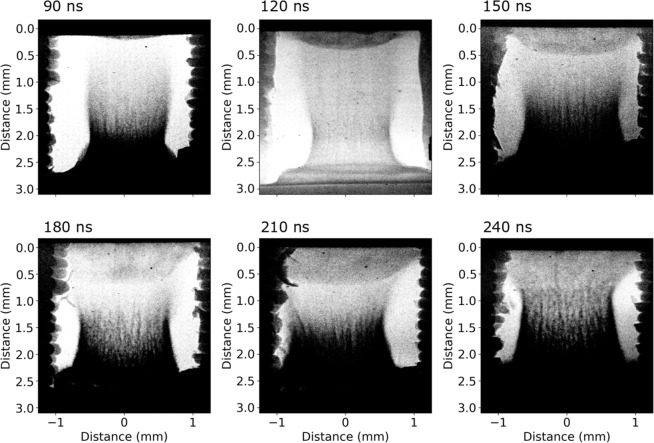
X-ray radiography images of plasma flow and reverse shock at different time delays. The laser driver interacts with the target at the bottom of the image creating a plasma flow moving upwards. The reverse shock initially moves downward away from the obstacle before reaching a stationary position beginning at 180 ns. The width of the reverse shock increases with time, indicating the presence of mass being evacuated in the lateral directions. The structures on the side of the images are not in the same plane as the experiment and hence do not play any role in the dynamics. Further details can be found in the Methods section.

The evolution of the reverse shock with time is visualised using X-ray radiography. Figure 5 shows six images over a range of 150 ns, spanning the formation and propagation of the reverse shock, all with an imposed B field of 15 T. The laser is incident from the bottom of the image, creating a plasma flow which travels upwards towards the obstacle. The reverse shock then moves counter to this flow, downwards on the image. The high temporal and spatial resolution of the data (10 ps, 25 μm, combined with the range of delay times measured, enable the detailed study of the physical processes at play in the post-shock region, inaccessible in previous work. Due to the demonstrable advantages of the magnetic field in reproducing the astrophysical case by collimating the plasma flow, as previously discussed, no data was taken at late times without the magnetic field.

## Discussion

We have already shown that the magnetic field helps to collimate to flow in the pre-shock or upstream region. In order to compare to the astrophysical case however, we now consider solely the post-shock or downstream region. The *β*_*ram*_ value (the ratio of thermal to magnetic pressure in a shocked system) is *β*_*ram*_ ~ 10^−2^–10^−4^ for the case of polars^10^ compared to ${\beta }_{ram}\lesssim 1$ for intermediate polars^34^. The huge magnetic field associated with polars means that strict Alfven similarity criteria cannot feasibly be met in a scaled laboratory experiment. However, with a value of *β*_*ram*_ ~ 1 in our experiment, we are in a regime that is similar to that of intermediate polars. We expect magnetic effects to play a significant (but not dominant) role in the dynamics of the system in the two cases. There is also good agreement in the magnetic Reynolds number, *R*_*m*_, (a measure of the diffusivity of the magnetic field inside the plasma), between the two systems (*R*_*m*_ ≫ 1 in MCVs and *R*_*m*_ ~ 10 in the laboratory). In both cases then the field lines are expected to be frozen in the magnetised plasma, with diffusive effects being small. The behaviour and predominant effect of the magnetic field in the two cases is expected to be the same; that is, the field is frozen in the plasma and will add an additional component to the Lorentz force, helping to collimate the flow, even in the post-shock region. Therefore, comparison between the two systems can be instructive.

**Figure 6:**
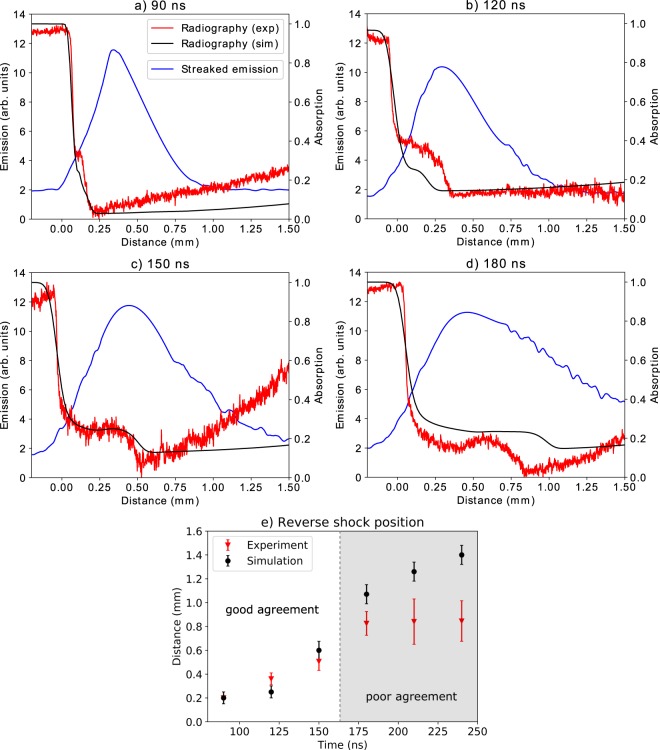
Lineouts taken from experimental and simulated X-ray radiographs as well as from the streaked optical emission diagnostic at four different times. The origin represents the obstacle vacuum interface, with positive values indicating the direction of propagation of the reverse shock. Also shown is the position of the reverse shock front as a function of time as measured by the simulations and experiment. The two cases begin to diverge from 180 ns onwards, with the experimental shock remaining stationary.

The propagation of the reverse shock is illustrated in Fig. 6 by taking 1-D lineouts from the emission and radiography images at different delays. Additionally, the experimental data are compared to synthetic X-ray radiographs, produced from 2D numerical magnetohydrodynamic (MHD) simulations, performed using the FLASH code (see Methods for more details). It is immediately obvious that the two experimental diagnostics disagree on the position of the reverse shock. At *t* ~ 90 ns (Fig. 6a), as the shock front is beginning to propagate away from the obstacle, one already observes a strong emission region further away from the obstacle. As time progresses, the dense shocked region moves at a faster speed than the emission region and therefore eventually catches up, whereupon it decelerates, before eventually stagnating at distance of ~0.8 mm away from the obstacle (see Fig. 6b–d). In both the simulated and experimental cases, before approaching the emission region (*t*  =  120 ns), the shock structure is indeed indicative of a regime where radiation plays a role, that is, the density is sloped downwards, and lacking a sharp discontinuity. Moreover, a sharp spike in the simulated electron temperature at the shock front is present, implying that radiative effects are present^35^. At later times, on the other hand, the picture changes. Indeed, in the experimental data, one observes the presence of a hollow region between the obstacle and the shock front, travelling away from the obstacle. Explicitly, this can be seen as a trough in the 1-D density lineout between the obstacle and the shock front, travelling away from the obstacle. This is typical of a rarefaction wave, caused by lateral mass ejection at the moment the incoming flow collides with the obstacle. This phenomenon is not present in the MHD simulations and so the reverse shock continues to propagate rather than becoming stationary, as seen in Fig. 6e. The increase in the radial extent of the reverse shock is also apparent in the X-ray radiography images in Fig. 5 with its width increasing from ~1 mm at 90 ns to ~1.6 m at 120 ns. Unfortunately it is not possible to measure the width quantitatively at later times as the target support blocks the line-of-sight of the X-rays. However, the effect is nonetheless apparent with a lateral extension of >2 mm at times later than 180 ns. Further images are shown in the Supplementary Information. The sound speed of the downstream plasma is estimated to be ~7 km/s, which is in agreement with the speed of the rarefaction wave observed in the experimental data, giving added support to our hypothesis that the simulation is unable to sufficiently capture the complex physics present in the collision region. Several astrophysical studies both concerning MCVs^36,37^ and accreting neutron stars^38,39^ have proposed the existence of hollow regions in the accretion column but observational evidence is lacking. The experimental results presented here may therefore provide valuable insights relevant for these theories. The mass loss rate, which has large implications for the density, temperature and emissivity of the post-shock region, is demonstrably poorly modelled within magnetohydrodynamic approximations. Similarly, standard 1D astrophysical models, where a cylindrical symmetry is assumed and matter flows along field lines, may be inadequate at describing accreting systems, with a full 3-D treatment necessary. There is currently no consensus, however, on how such a 3-D model might be constructed^40–42^. Open questions remain concerning the role of the laterally ejected matter, including whether it is eventually absorbed by the WD or is able to escape, and whether it plays a significant role in the energy balance of the system^14^. The expansion of the lower part of accretion columns has also been linked to the poorly understood oscillatory nature of the luminosity of polars^18^, and further study is required to investigate this phenomenon. This experiment therefore paves the way for future studies with higher magnetic fields (30–40 T) and at larger laser facilities, able to create plasma systems where radiation dominates, and to achieve full similarity to the astrophysical case. This line of research therefore represents a tangible way to distinguish between a wide range of unconfirmed astrophysical models and to resolve a variety of outstanding uncertainties surrounding MCVs.

To conclude, we compare the shock standoff distance observed in the laboratory to those taken from a typical astrophysical accretion column for the specific case of a an intermediate polar, by employing the scaling method as described in^43,44^. The values relevant to the scaling between the two systems are summarized in Table 1. The astrophysical system has a characteristic cooling time and velocity of, *t*_*cool*_ = 1 s and *v* = 1000 km/s respectively. The cooling time in the experimental case is calculated using two different methods. The first is using the formula: *t*_*cool*_ = *P*/$\epsilon$ (*γ* − 1), where *P* is the pressure of the post-shock region, and $\epsilon$, the emissivity, is related to the Planck mean free path, *λ*_*pl*_ via the relation $\epsilon$ = *σT*^4^/*λ*_*pl*_. The second method follows the formulation proposed by^45^, for the case of a black body. The two approaches are approximately in agreement, giving 3 and 4 ns respectively. Taking the measured experimental velocity of *v* = 75 km/s and shock standoff distance of *h*_*s*_ = 0.8 ± 0.1 mm, we then calculate a corresponding distance of 2000–3000 km for the astrophysical regime. This value is of the same order of magnitude to those predicted by theory^12^, although is slightly larger than previous experimental results^29^. This discrepancy once again underlines the failings of simple 1-D models as well as the limitations of previous experiments, and provides further evidence of the need to model accretion columns in a fully 3-D manner.

**Table 1:** Scaling between the laboratory system and a typical intermediate polar.

Parameter	Lab	Astro
Plasma *β*_*ram*_	1	$\lesssim 1$
Magnetic Reynolds number, *R*_*m*_	10	≫ 1
Fluid Reynolds number, *R*	10^5^	10^6^
Mach number, *M*	2	>10
Cooling time, *t*_*cool*_	3–4 ns	~1 s
Cooling parameter, *χ*	0.5	≪1
Shock standoff distance, *h*_*s*_,	0.8 ± 0.1 mm	~1000 km

The cooling parameter is defined as the ratio of the cooling time to the characteristic hydrodynamical timescale *χ* = *t*_*cool*_/*t*_*hyd*_ and is a measure of the importance of radiative effects. Values for the astrophysical system are taken from^13,27^.

This work builds upon the previously established platform to study the physics of accretion processes in magnetic cataclysmic variables, currently inaccessible with observations. The quality and wealth of radiographic and optical data allow us to fully understand the dynamics of the laboratory system for the first time, revealing a complex interplay of material, thermal, radiative and magnetic effects, previously unseen in experiments. The results reveal that clear improvements are made by the deployment of the external magnetic field over a plastic tube or simple inertial collimation. By measuring a steady shock front over an extended period of time, we are able to scale the experimental regime to the astrophysical one, gaining further insight into the complex dynamics at play in both systems. In particular, we observe a significant mass ejection in the transverse direction at the moment the plasma flow impacts upon the obstacle, not predicted by MHD simulations. This changes the structure of the post shock region and has important implications for astrophysical models, including those used to determine, for example, the WD’s mass. A clear pathway for future experiments, exploring a radiation dominated regime exists is therefore apparent; this could be achieved, for example, on magnetic field platforms at facilities such as the NIF or LMJ where higher laser energies are available. In this manner, astrophysical accretion models may be refined and uncertainties surrounding these systems may finally be resolved.

## Methods

### Experimental platform

The experiment was carried out at the LULI2000 laser facility at the LULI laboratory (Ecole Polytechnique, France). A long pulse (*t* = 1.5 ns), high energy (*E* = 500 J, *λ* = 527 nm) drive beam was used to produce a strong shock wave in a solid multi-layer target. The target consisted of a very thin (several nm) layer of aluminium on the laser facing side, attached to a 25 μm layer of ablator material (CH), a 1.5 μm layer of gold to act as a radiation shield produced by the corona, and finally a 6 μm layer of titanium. The target was fixed onto a 4 mm diameter holder with a 2 mm diameter inner hole. A flat-topped 500 μm focal spot was produced using a hybrid phase plate. The plasma flow produced by the shock breakout at the rear surface of the target impacted onto a gadolinium gallium garnet (GGG) obstacle (chosen for its transparency and high density, *ρ* = 7.08 g/cm^3^) at a distance of 3 mm.

### Magnetic field

An external magnetic field of 15 T, generated by a specially designed coil, coupled to a pulsed power generator, is applied to the whole experimental system. The capacitor-based pulse generator is charged up to a voltage of 9.6 kV and provides a peak current of 23.6 kA to the coil. The magnetic field inside the coil reaches its peak value after 183 μs and stays constant (less than 2% variation) over a duration of microseconds; much longer than the timescales of the experiment. The drive lasers were fired when the magnetic field reached its maximum value.

### X-ray diagnostics

The X-ray source was generated by the interaction of a high-intensity, short-pulse laser (50 J, 10 ps, with a focal spot of 50 μm) and a titanium wire target. The wire was positioned 3 cm below the main target and the image was recorded onto an imaging plate 60 cm above the experimental plane, giving a magnification factor of 20^33^.

### Optical diagnostics

A probe laser beam (1 mJ, 7 ns, *λ* = 532 nm) was used to produce Schlieren images of the plasma at various delay times. The images were recorded using CCDs, coupled to gated optical imagers (GOIs) with a 200 ps gate time. Optical self-emission of the experimental region was also recorded at a wavelength of 450 ± 40 nm using a streak camera. Both diagnostics give the speed of the plasma flow as well as relative density and temperature measurements respectively.

### Numerical simulations

The numerical simulations were performed using the FLASH code, developed at the University of Chicago. A non-ideal MHD solver with an unsplit staggered mesh was used together with physical modules that allow modeling high energy density laser experiments including a laser energy deposition module, SESAME equations of state and radiation transfer, solved in the multi-group diffusion approximation using 40 radiation groups. A constant magnetic field of 15 T was applied in the direction of the propagation of the flow. The laser intensity focal spot on target was a super Gaussian function with a diameter of 500 μm. The rise time of the laser beam was 0.2 ns, followed by a plateau at maximum intensity of 1.7 × 10^14^ W cm^−2^ for a period of 1.3 ns. The simulation had a resolution of 5.12 μm and hence the thin layer of gold in the multi-layer target was not able to be resolved. A mass-density equivalent metal target layer was therefore used. The X-ray radiographs were produced assuming a quasi-monochromatic Ti backlighter source at 4.51 keV and using cold opacities^46^. Additional figures are shown in the Supplementary Information.

## Supplementary information

Supplementary Information

## Data Availability

The authors declare that the data supporting the findings of this study are available from the authors upon request.
