# Supplementary material for: Laboratory study of stationary accretion shock relevant to astrophysical systems
Source: Sci Rep. 2019 May 31;9:8157. doi: 10.1038/s41598-019-44596-3 (PMC6544622; doi:10.1038/s41598-019-44596-3)
Supplement: Supplementary file 1 — Supplementary Information [file 41598_2019_44596_MOESM1_ESM.pdf]

Supplementary Information

Laboratory study of stationary accretion shock relevant to astrophysical systems

P. Mabey[1], B. Albertazzi[1], E. Falize[2,3], Th. Michel[1], G. Rigon[1], L. Van Box Som[2,3], A. Pelka[4], F,-E. Brack[4,5], F. Kroll[4], E. Filippov[6,7], G. Gregori[8], Y. Kuramitsu[9,10], D. Q. Lamb[1]1, C. Li[12], N. Ozaki[9], S. Pikuz[6,7], Y. Sakawa[13], P. Tzeferacos[8,11], M. Koenig[1,9]

1. LULI - CNRS, Ecole Polytechnique, CEA, Université Paris-Saclay, F-91128 Palaiseau Cedex, France
2. CEA-DAM-DIF, F-91297 Arpajon, France
3. CEA Saclay, DSM/Irfu/Service d'Astrophysique, F-91191 Gif-sur-Yvette, France
4. Helmholtz-Zentrum Dresden-Rossendorf (HZDR), Bautzner Landstr. 400, D-01328 Dresden, Germany
5. Technische Universität Dresden, D-01062 Dresden, Germany
6. JIHT-RAS, 13-2 Izhorskaya st., Moscow, 125412 Russia
7. National Research Nuclear University MEPhI, Moscow 115409, Russia
8. Department of Physics, University of Oxford, Parks Road, Oxford OX1 3PU, UK
9. Graduate School of Engineering, Osaka University, Suita, Osaka 565-0871, Japan
10. Department of Physics, National Central University, Taoyuan City, Taiwan
11. Department of Astronomy and Astrophysics, University of Chicago, Chicago, IL, USA
12. Plasma Science and Fusion Center, Massachusetts Institute of Technology, 77 Massachusetts Avenue, Cambridge, MA 02139 USA
13. Institute of Laser Engineering, Osaka University, Suita, Osaka 565-0871, Japan

The following three figures represent results which are not integral to understanding the work presented in the main text. They are presented here in order to substantiate some of the claims made in the paper and to give the reader a greater understanding of the results. In all images, the laser is incident from below, causing the plasma flow to move upwards towards the obstacle. Fig. 1 shows the synthetic X-ray radiographs against the experimental images. For early times, the two are visibly strikingly similar whereas at late times the reverse shock is noticeably more efficiently collimated and has travelled further in the simulated case. One of the central claims of the paper is the evidence of an increased width of the dense post-shock region, so-called lateral mass ejection. Fig. 2 shows an enlarged image of this region, with the contrast enhanced for the sake of clarity. As mentioned in the main text, the support, the shadow of which is seen on the right hand side of the image, prevents the quantitative measurement of the width of the post-shock region at late times. Since only relative temperature and density measurements were possible experimentally, and no absolute values were possible, the dimensionless plasma parameters discussed in the main text are calculated from the simulated values shown in fig. 3. The similarity between the experimental results and the simulated ones shown in fig. 1 gives us confidence that these values are indeed accurate.

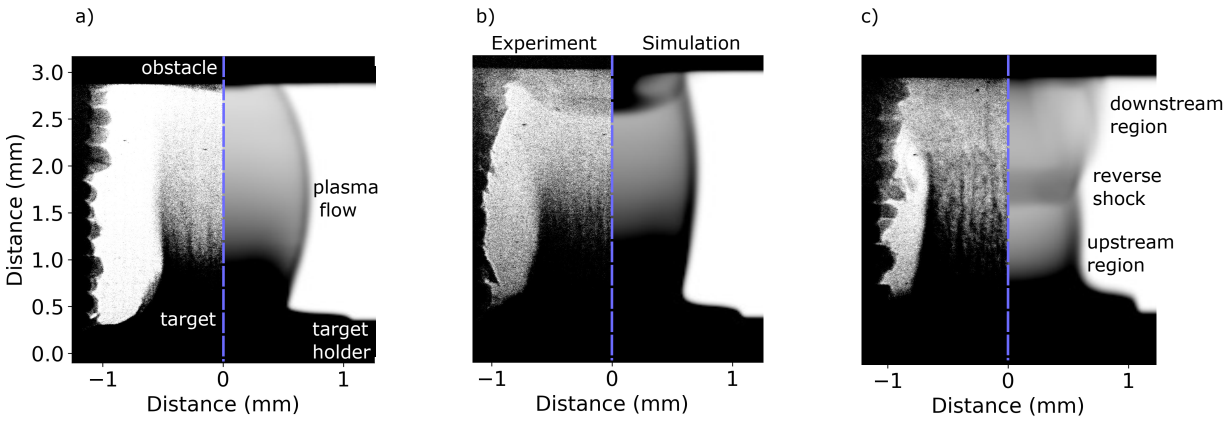

Figure 1: Comparison of FLASH simulations and experimental X-ray radiography data at (left) 90 ns, (center) 150 ns and (right) 240 ns. The simulated results are blurred using a Gaussian of width 25 µm in order to reproduce the resolution of the experimental setup.

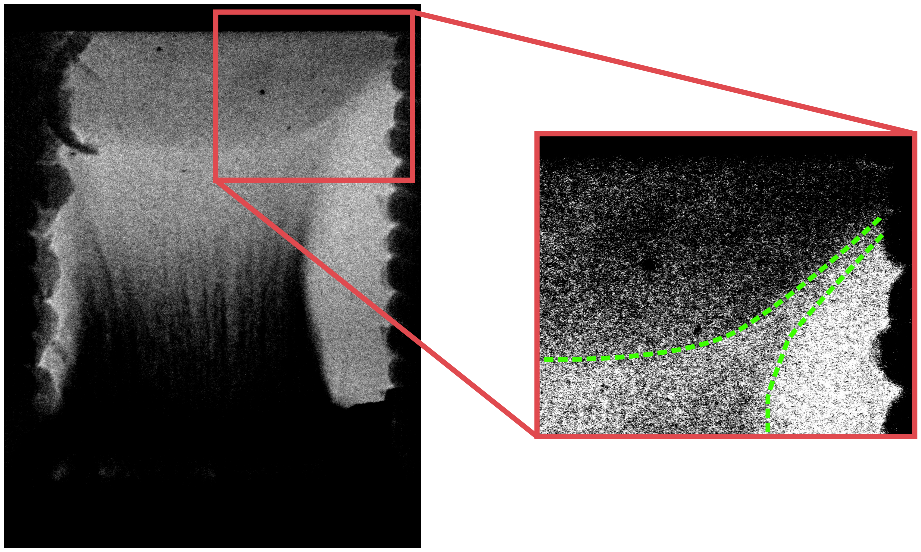

Figure 2: Experimental X-ray radiography image at 210 ns. Inset shows enlarged image of collision region showing increase in jet width with mass being ejected in the lateral direction. Contrast is enhanced in the zoomed image to aid visibility.

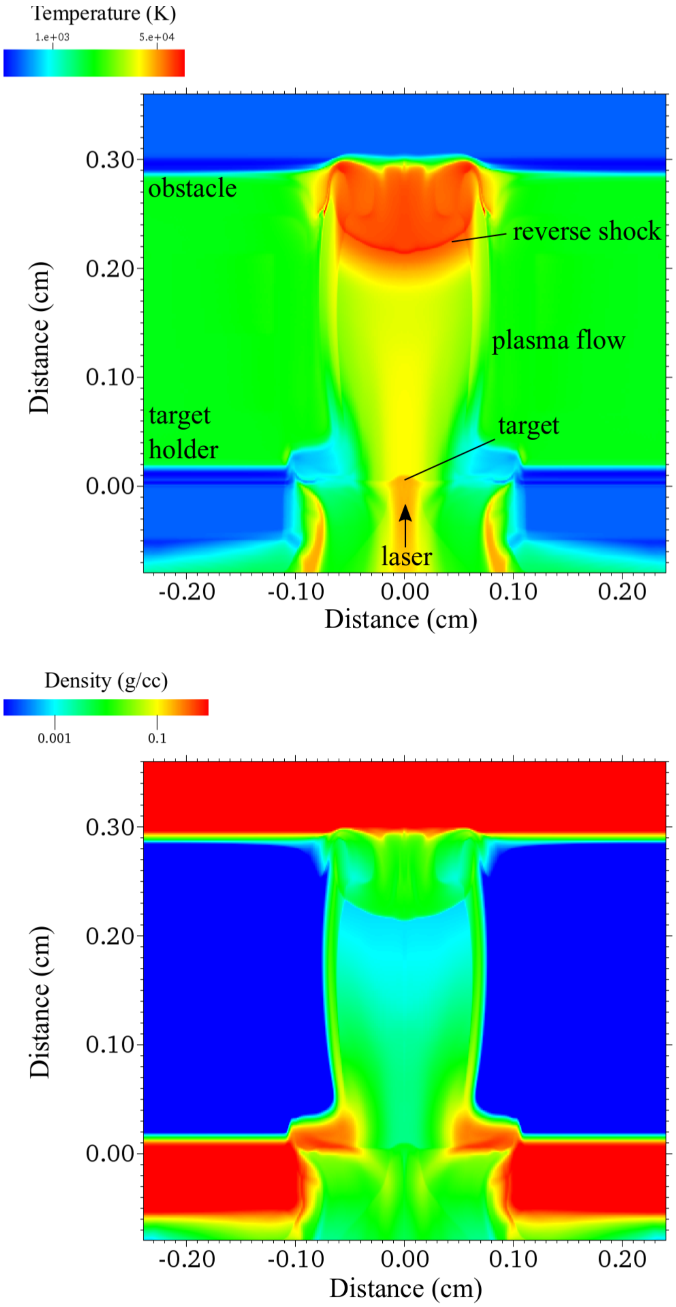

Figure 3: Temperature and mass density plots taken from FLASH simulations at 150 ns. The values within are used for various calculations in the main text.
